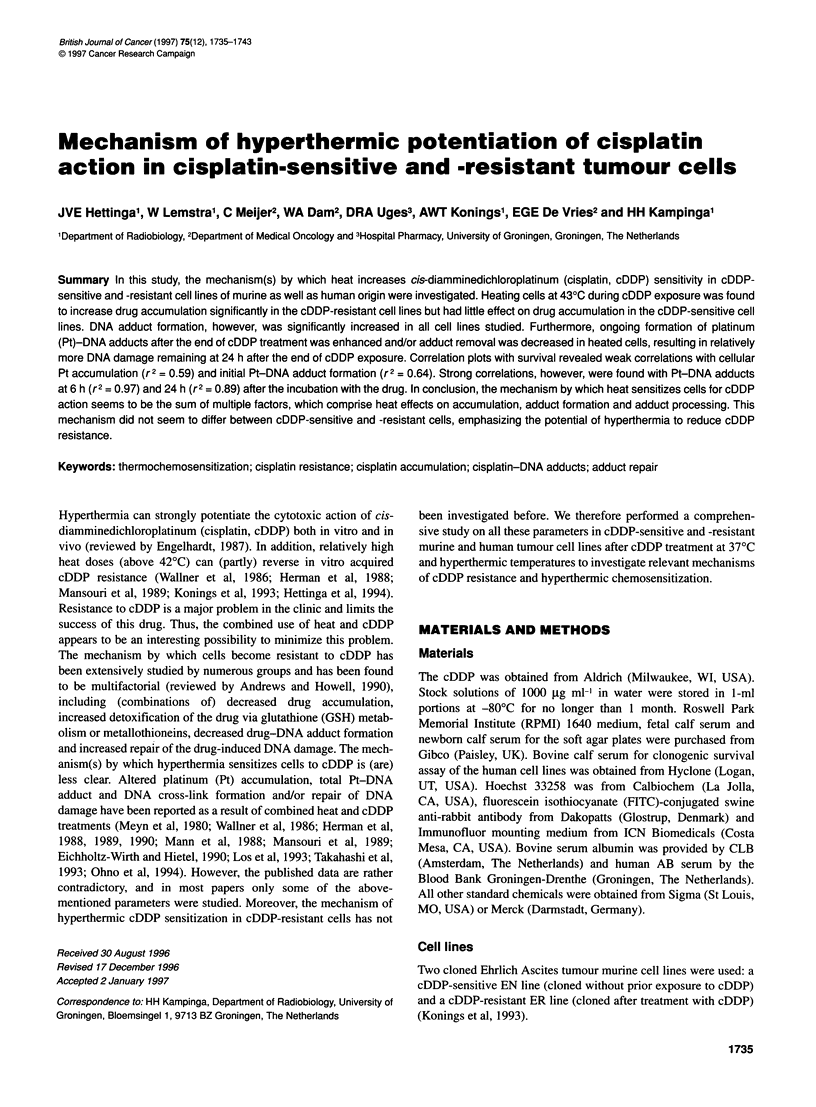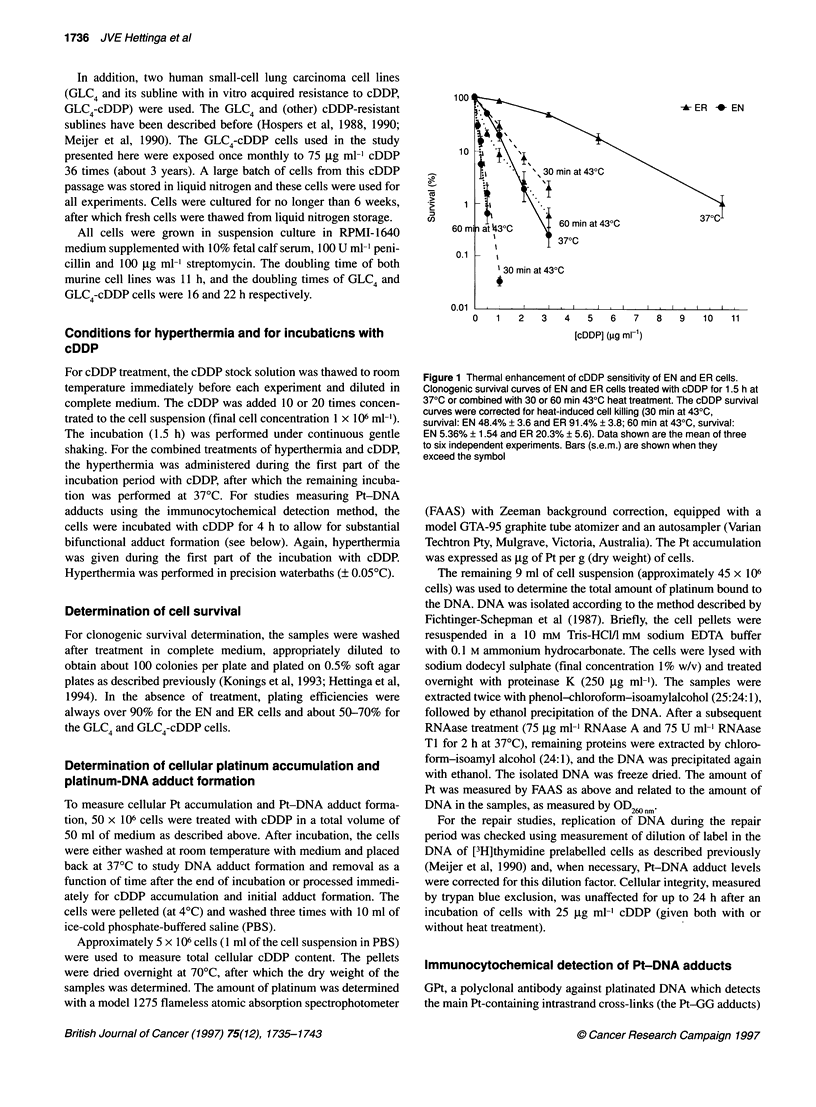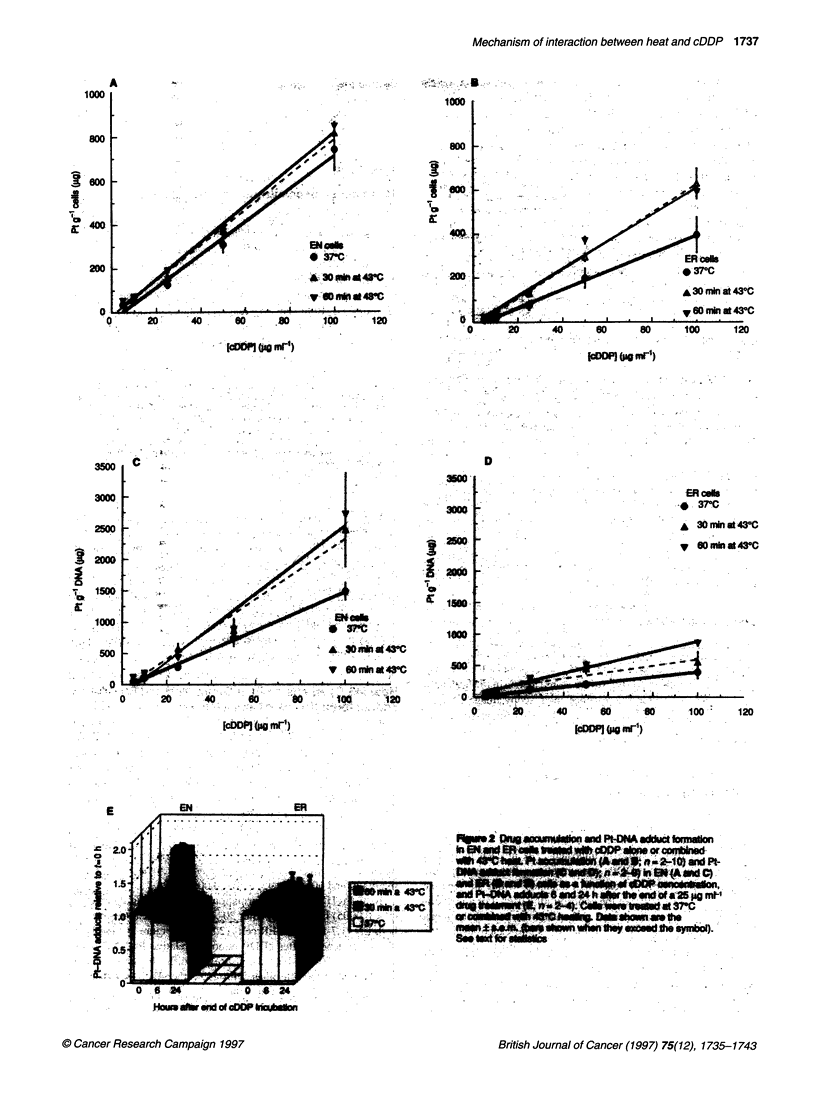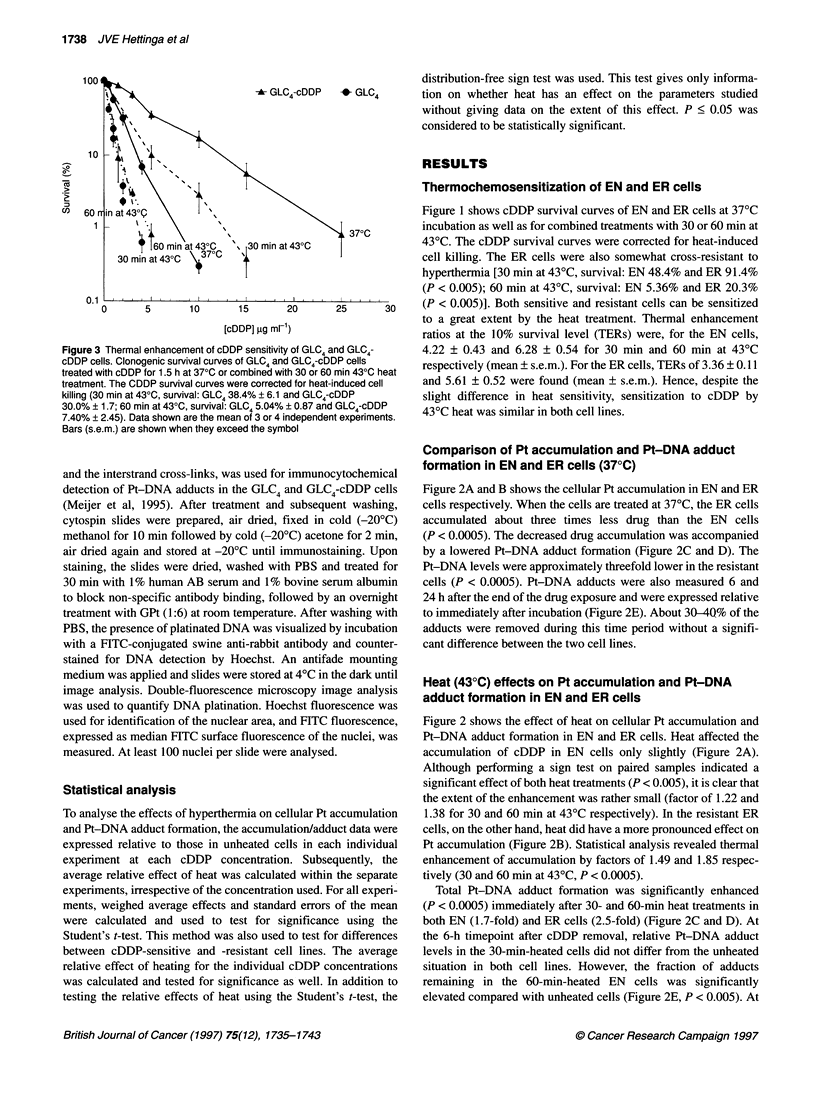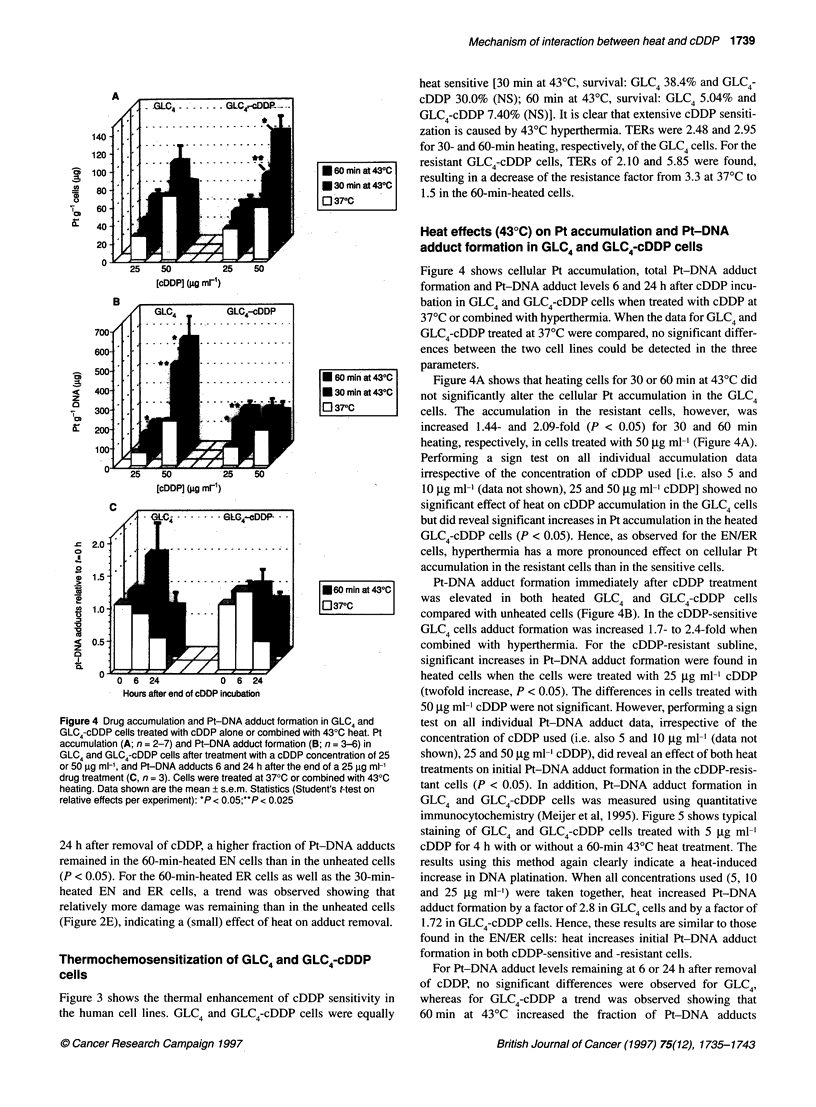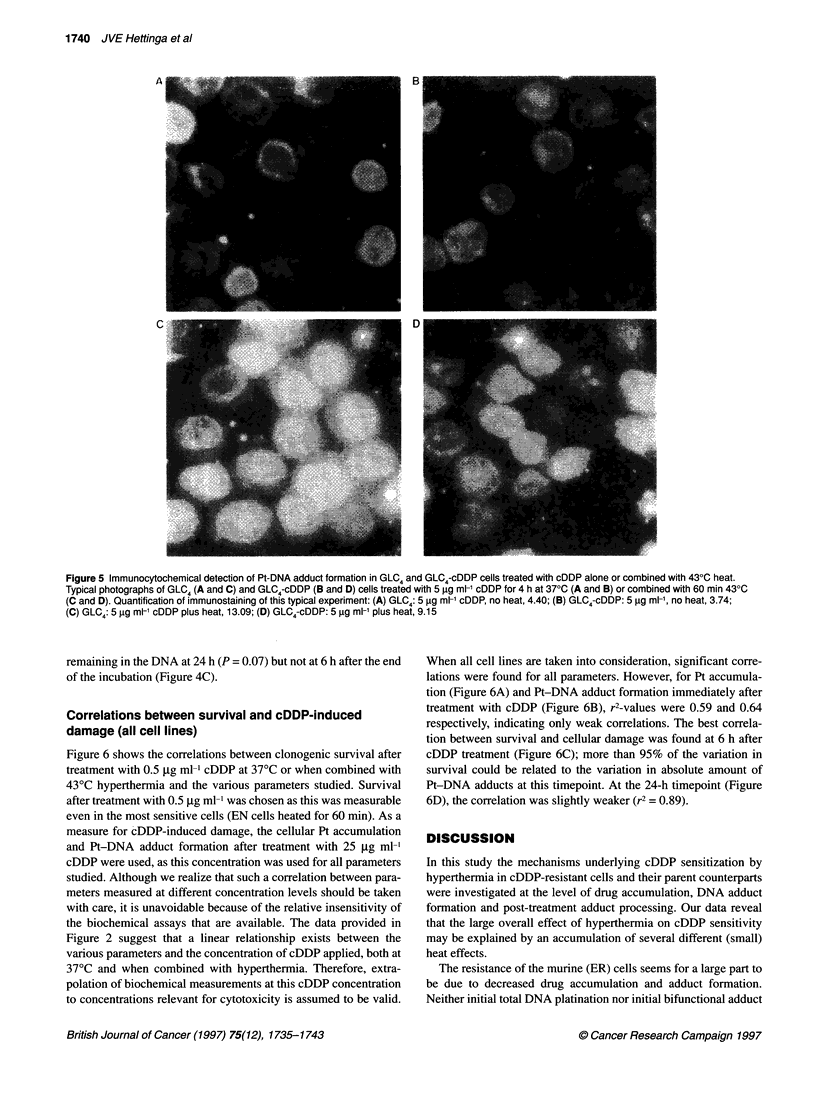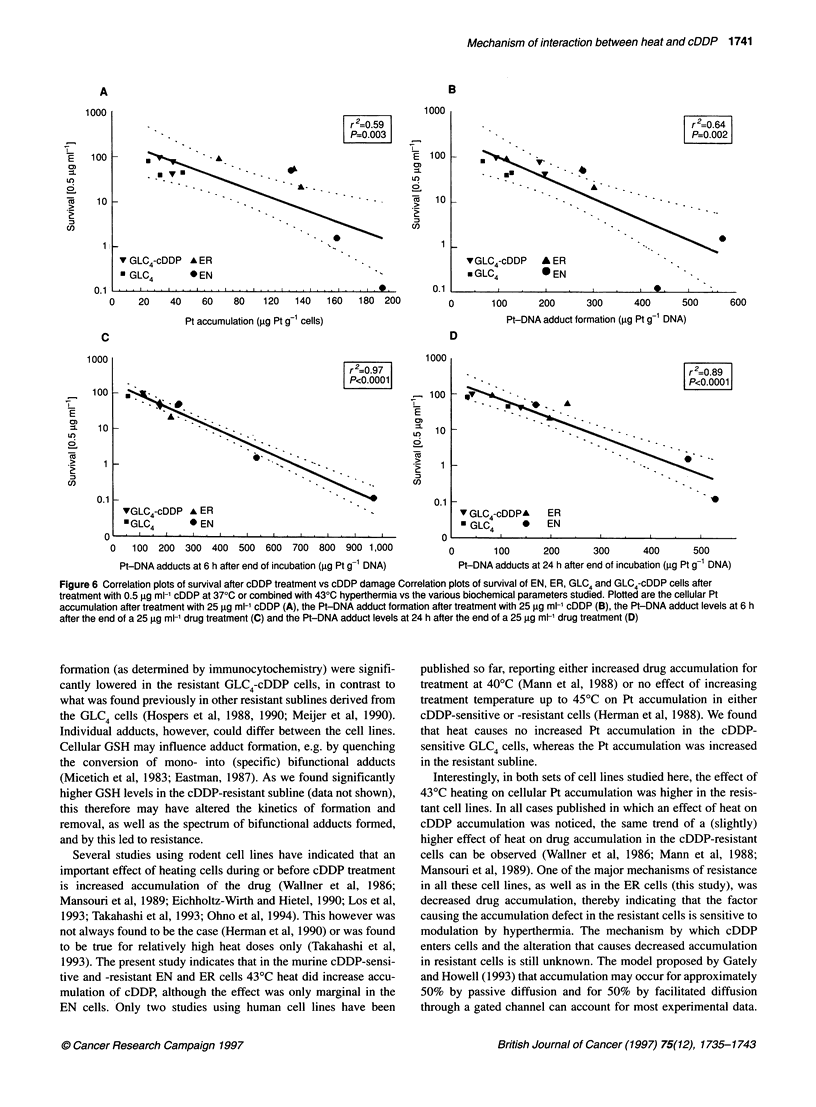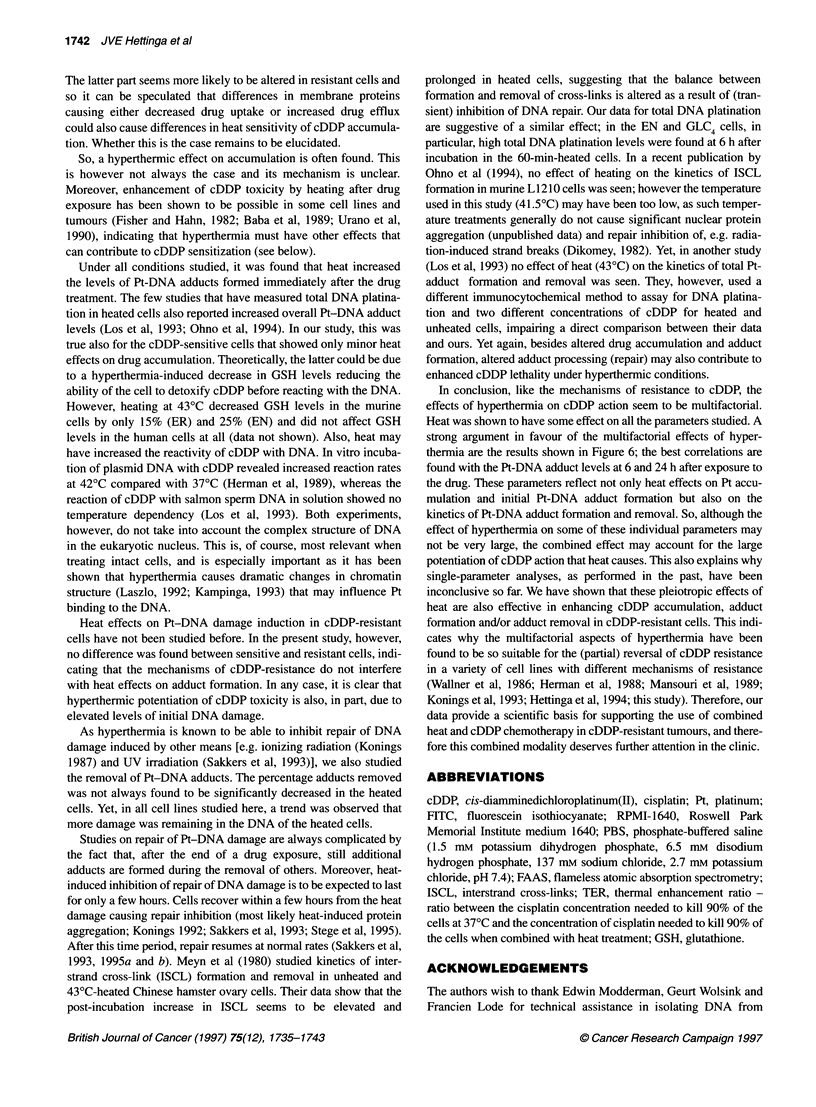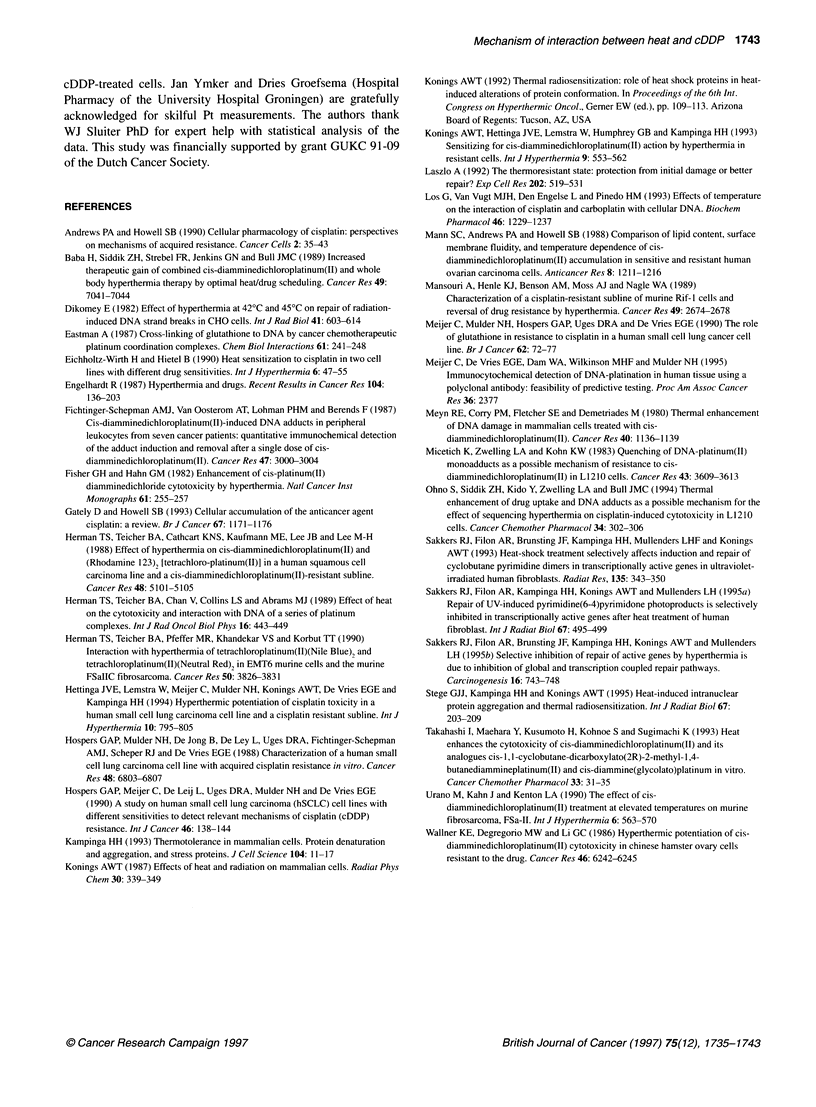# Mechanism of hyperthermic potentiation of cisplatin action in cisplatin-sensitive and -resistant tumour cells.

**DOI:** 10.1038/bjc.1997.297

**Published:** 1997

**Authors:** J. V. Hettinga, W. Lemstra, C. Meijer, W. A. Dam, D. R. Uges, A. W. Konings, E. G. De Vries, H. H. Kampinga

**Affiliations:** Department of Radiobiology, University of Groningen, The Netherlands.

## Abstract

**Images:**


British Journal of Cancer (1997) 75(12), 1735–1743
© 1997 Cancer Research Campaign

### Mechanism of hyperthermic potentiation of cisplatin action in cisplatin-sensitive and -resistant tumour cells

**JVE Hettinga[1], W Lemstra[1], C Meijer[2], WA Dam[2], DRA Uges[3], AWT Konings[1], EGE De Vries[2] and HH Kampinga[1]**

[1]Department of Radiobiology, [2]Department of Medical Oncology and [3]Hospital Pharmacy, University of Groningen, Groningen, The Netherlands

**Summary** In this study, the mechanism(s) by which heat increases *cis*-diamminedichloroplatinum (cisplatin, cDDP) sensitivity in cDDP-sensitive and -resistant cell lines of murine as well as human origin were investigated. Heating cells at 43°C during cDDP exposure was found to increase drug accumulation significantly in the cDDP-resistant cell lines but had little effect on drug accumulation in the cDDP-sensitive cell lines. DNA adduct formation, however, was significantly increased in all cell lines studied. Furthermore, ongoing formation of platinum (Pt)–DNA adducts after the end of cDDP treatment was enhanced and/or adduct removal was decreased in heated cells, resulting in relatively more DNA damage remaining at 24 h after the end of cDDP exposure. Correlation plots with survival revealed weak correlations with cellular Pt accumulation ($r^2$ = 0.59) and initial Pt–DNA adduct formation ($r^2$ = 0.64). Strong correlations, however, were found with Pt–DNA adducts at 6 h ($r^2$ = 0.97) and 24 h ($r^2$ = 0.89) after the incubation with the drug. In conclusion, the mechanism by which heat sensitizes cells for cDDP action seems to be the sum of multiple factors, which comprise heat effects on accumulation, adduct formation and adduct processing. This mechanism did not seem to differ between cDDP-sensitive and -resistant cells, emphasizing the potential of hyperthermia to reduce cDDP resistance.

**Keywords:** thermochemosensitization; cisplatin resistance; cisplatin accumulation; cisplatin–DNA adducts; adduct repair

Hyperthermia can strongly potentiate the cytotoxic action of *cis*-diamminedichloroplatinum (cisplatin, cDDP) both in vitro and in vivo (reviewed by Engelhardt, 1987). In addition, relatively high heat doses (above 42°C) can (partly) reverse in vitro acquired cDDP resistance (Wallner et al, 1986; Herman et al, 1988; Mansouri et al, 1989; Konings et al, 1993; Hettinga et al, 1994). Resistance to cDDP is a major problem in the clinic and limits the success of this drug. Thus, the combined use of heat and cDDP appears to be an interesting possibility to minimize this problem. The mechanism by which cells become resistant to cDDP has been extensively studied by numerous groups and has been found to be multifactorial (reviewed by Andrews and Howell, 1990), including (combinations of) decreased drug accumulation, increased detoxification of the drug via glutathione (GSH) metabolism or metallothioneins, decreased drug–DNA adduct formation and increased repair of the drug-induced DNA damage. The mechanism(s) by which hyperthermia sensitizes cells to cDDP is (are) less clear. Altered platinum (Pt) accumulation, total Pt–DNA adduct and DNA cross-link formation and/or repair of DNA damage have been reported as a result of combined heat and cDDP treatments (Meyn et al, 1980; Wallner et al, 1986; Herman et al, 1988, 1989, 1990; Mann et al, 1988; Mansouri et al, 1989; Eichholtz-Wirth and Hietel, 1990; Los et al, 1993; Takahashi et al, 1993; Ohno et al, 1994). However, the published data are rather contradictory, and in most papers only some of the above-mentioned parameters were studied. Moreover, the mechanism of hyperthermic cDDP sensitization in cDDP-resistant cells has not been investigated before. We therefore performed a comprehensive study on all these parameters in cDDP-sensitive and -resistant murine and human tumour cell lines after cDDP treatment at 37°C and hyperthermic temperatures to investigate relevant mechanisms of cDDP resistance and hyperthermic chemosensitization.

*Received 30 August 1996*
*Revised 17 December 1996*
*Accepted 2 January 1997*

*Correspondence to:* HH Kampinga, Department of Radiobiology, University of Groningen, Bloemsingel 1, 9713 BZ Groningen, The Netherlands

### MATERIALS AND METHODS

### Materials

The cDDP was obtained from Aldrich (Milwaukee, WI, USA). Stock solutions of 1000 μg $ml^{-1}$ in water were stored in 1-ml portions at –80°C for no longer than 1 month. Roswell Park Memorial Institute (RPMI) 1640 medium, fetal calf serum and newborn calf serum for the soft agar plates were purchased from Gibco (Paisley, UK). Bovine calf serum for clonogenic survival assay of the human cell lines was obtained from Hyclone (Logan, UT, USA). Hoechst 33258 was from Calbiochem (La Jolla, CA, USA), fluorescein isothiocyanate (FITC)-conjugated swine anti-rabbit antibody from Dakopatts (Glostrup, Denmark) and Immunofluor mounting medium from ICN Biomedicals (Costa Mesa, CA, USA). Bovine serum albumin was provided by CLB (Amsterdam, The Netherlands) and human AB serum by the Blood Bank Groningen-Drenthe (Groningen, The Netherlands). All other standard chemicals were obtained from Sigma (St Louis, MO, USA) or Merck (Darmstadt, Germany).

### Cell lines

Two cloned Ehrlich Ascites tumour murine cell lines were used: a cDDP-sensitive EN line (cloned without prior exposure to cDDP) and a cDDP-resistant ER line (cloned after treatment with cDDP) (Konings et al, 1993).

In addition, two human small-cell lung carcinoma cell lines ($GLC_4$ and its subline with in vitro acquired resistance to cDDP, $GLC_4$-cDDP) were used. The $GLC_4$ and (other) cDDP-resistant sublines have been described before (Hospers et al, 1988, 1990; Meijer et al, 1990). The $GLC_4$-cDDP cells used in the study presented here were exposed once monthly to 75 μg ml$^{-1}$ cDDP 36 times (about 3 years). A large batch of cells from this cDDP passage was stored in liquid nitrogen and these cells were used for all experiments. Cells were cultured for no longer than 6 weeks, after which fresh cells were thawed from liquid nitrogen storage.

All cells were grown in suspension culture in RPMI-1640 medium supplemented with 10% fetal calf serum, 100 U ml$^{-1}$ penicillin and 100 μg ml$^{-1}$ streptomycin. The doubling time of both murine cell lines was 11 h, and the doubling times of $GLC_4$ and $GLC_4$-cDDP cells were 16 and 22 h respectively.

### Conditions for hyperthermia and for incubations with cDDP

For cDDP treatment, the cDDP stock solution was thawed to room temperature immediately before each experiment and diluted in complete medium. The cDDP was added 10 or 20 times concentrated to the cell suspension (final cell concentration 1 × 10$^6$ ml$^{-1}$). The incubation (1.5 h) was performed under continuous gentle shaking. For the combined treatments of hyperthermia and cDDP, the hyperthermia was administered during the first part of the incubation period with cDDP, after which the remaining incubation was performed at 37°C. For studies measuring Pt–DNA adducts using the immunocytochemical detection method, the cells were incubated with cDDP for 4 h to allow for substantial bifunctional adduct formation (see below). Again, hyperthermia was given during the first part of the incubation with cDDP. Hyperthermia was performed in precision waterbaths (± 0.05°C).

### Determination of cell survival

For clonogenic survival determination, the samples were washed after treatment in complete medium, appropriately diluted to obtain about 100 colonies per plate and plated on 0.5% soft agar plates as described previously (Konings et al, 1993; Hettinga et al, 1994). In the absence of treatment, plating efficiencies were always over 90% for the EN and ER cells and about 50–70% for the $GLC_4$ and $GLC_4$-cDDP cells.

### Determination of cellular platinum accumulation and platinum-DNA adduct formation

To measure cellular Pt accumulation and Pt–DNA adduct formation, 50 × 10$^6$ cells were treated with cDDP in a total volume of 50 ml of medium as described above. After incubation, the cells were either washed at room temperature with medium and placed back at 37°C to study DNA adduct formation and removal as a function of time after the end of incubation or processed immediately for cDDP accumulation and initial adduct formation. The cells were pelleted (at 4°C) and washed three times with 10 ml of ice-cold phosphate-buffered saline (PBS).

Approximately 5 × 10$^6$ cells (1 ml of the cell suspension in PBS) were used to measure total cellular cDDP content. The pellets were dried overnight at 70°C, after which the dry weight of the samples was determined. The amount of platinum was determined with a model 1275 flameless atomic absorption spectrophotometer

**Figure 1** Thermal enhancement of cDDP sensitivity of EN and ER cells. Clonogenic survival curves of EN and ER cells treated with cDDP for 1.5 h at 37°C or combined with 30 or 60 min 43°C heat treatment. The cDDP survival curves were corrected for heat-induced cell killing (30 min at 43°C, survival: EN 48.4% ± 3.6 and ER 91.4% ± 3.8; 60 min at 43°C, survival: EN 5.36% ± 1.54 and ER 20.3% ± 5.6). Data shown are the mean of three to six independent experiments. Bars (s.e.m.) are shown when they exceed the symbol

(FAAS) with Zeeman background correction, equipped with a model GTA-95 graphite tube atomizer and an autosampler (Varian Techtron Pty, Mulgrave, Victoria, Australia). The Pt accumulation was expressed as μg of Pt per g (dry weight) of cells.

The remaining 9 ml of cell suspension (approximately 45 × 10$^6$ cells) was used to determine the total amount of platinum bound to the DNA. DNA was isolated according to the method described by Fichtinger-Schepman et al (1987). Briefly, the cell pellets were resuspended in a 10 mM Tris-HCl/1 mM sodium EDTA buffer with 0.1 M ammonium hydrocarbonate. The cells were lysed with sodium dodecyl sulphate (final concentration 1% w/v) and treated overnight with proteinase K (250 μg ml$^{-1}$). The samples were extracted twice with phenol–chloroform–isoamylalcohol (25:24:1), followed by ethanol precipitation of the DNA. After a subsequent RNAase treatment (75 μg ml$^{-1}$ RNAase A and 75 U ml$^{-1}$ RNAase T1 for 2 h at 37°C), remaining proteins were extracted by chloroform–isoamyl alcohol (24:1), and the DNA was precipitated again with ethanol. The isolated DNA was freeze dried. The amount of Pt was measured by FAAS as above and related to the amount of DNA in the samples, as measured by $OD_{260\,nm}$.

For the repair studies, replication of DNA during the repair period was checked using measurement of dilution of label in the DNA of [$^3$H]thymidine prelabelled cells as described previously (Meijer et al, 1990) and, when necessary, Pt–DNA adduct levels were corrected for this dilution factor. Cellular integrity, measured by trypan blue exclusion, was unaffected for up to 24 h after an incubation of cells with 25 μg ml$^{-1}$ cDDP (given both with or without heat treatment).

### Immunocytochemical detection of Pt–DNA adducts

GPt, a polyclonal antibody against platinated DNA which detects the main Pt-containing intrastrand cross-links (the Pt–GG adducts)

© Cancer Research Campaign 1997

**Figure 3** Thermal enhancement of cDDP sensitivity of $GLC_4$ and $GLC_4$-cDDP cells. Clonogenic survival curves of $GLC_4$ and $GLC_4$-cDDP cells treated with cDDP for 1.5 h at 37°C or combined with 30 or 60 min 43°C heat treatment. The CDDP survival curves were corrected for heat-induced cell killing (30 min at 43°C, survival: $GLC_4$ 38.4% ± 6.1 and $GLC_4$-cDDP 30.0% ± 1.7; 60 min at 43°C, survival: $GLC_4$ 5.04% ± 0.87 and $GLC_4$-cDDP 7.40% ± 2.45). Data shown are the mean of 3 or 4 independent experiments. Bars (s.e.m.) are shown when they exceed the symbol and the interstrand cross-links, was used for immunocytochemical detection of Pt–DNA adducts in the $GLC_4$ and $GLC_4$-cDDP cells (Meijer et al, 1995). After treatment and subsequent washing, cytospin slides were prepared, air dried, fixed in cold (–20°C) methanol for 10 min followed by cold (–20°C) acetone for 2 min, air dried again and stored at –20°C until immunostaining. Upon staining, the slides were dried, washed with PBS and treated for 30 min with 1% human AB serum and 1% bovine serum albumin to block non-specific antibody binding, followed by an overnight treatment with GPt (1:6) at room temperature. After washing with PBS, the presence of platinated DNA was visualized by incubation with a FITC-conjugated swine anti-rabbit antibody and counterstained for DNA detection by Hoechst. An antifade mounting medium was applied and slides were stored at 4°C in the dark until image analysis. Double-fluorescence microscopy image analysis was used to quantify DNA platination. Hoechst fluorescence was used for identification of the nuclear area, and FITC fluorescence, expressed as median FITC surface fluorescence of the nuclei, was measured. At least 100 nuclei per slide were analysed.

### Statistical analysis

To analyse the effects of hyperthermia on cellular Pt accumulation and Pt–DNA adduct formation, the accumulation/adduct data were expressed relative to those in unheated cells in each individual experiment at each cDDP concentration. Subsequently, the average relative effect of heat was calculated within the separate experiments, irrespective of the concentration used. For all experiments, weighed average effects and standard errors of the mean were calculated and used to test for significance using the Student's $t$-test. This method was also used to test for differences between cDDP-sensitive and -resistant cell lines. The average relative effect of heating for the individual cDDP concentrations was calculated and tested for significance as well. In addition to testing the relative effects of heat using the Student's $t$-test, the distribution-free sign test was used. This test gives only information on whether heat has an effect on the parameters studied without giving data on the extent of this effect. $P \leq 0.05$ was considered to be statistically significant.

### RESULTS

### Thermochemosensitization of EN and ER cells

Figure 1 shows cDDP survival curves of EN and ER cells at 37°C incubation as well as for combined treatments with 30 or 60 min at 43°C. The cDDP survival curves were corrected for heat-induced cell killing. The ER cells were also somewhat cross-resistant to hyperthermia [30 min at 43°C, survival: EN 48.4% and ER 91.4% ($P < 0.005$); 60 min at 43°C, survival: EN 5.36% and ER 20.3% ($P < 0.005$)]. Both sensitive and resistant cells can be sensitized to a great extent by the heat treatment. Thermal enhancement ratios at the 10% survival level (TERs) were, for the EN cells, 4.22 ± 0.43 and 6.28 ± 0.54 for 30 min and 60 min at 43°C respectively (mean ± s.e.m.). For the ER cells, TERs of 3.36 ± 0.11 and 5.61 ± 0.52 were found (mean ± s.e.m.). Hence, despite the slight difference in heat sensitivity, sensitization to cDDP by 43°C heat was similar in both cell lines.

### Comparison of Pt accumulation and Pt–DNA adduct formation in EN and ER cells (37°C)

Figure 2A and B shows the cellular Pt accumulation in EN and ER cells respectively. When the cells are treated at 37°C, the ER cells accumulated about three times less drug than the EN cells ($P < 0.0005$). The decreased drug accumulation was accompanied by a lowered Pt–DNA adduct formation (Figure 2C and D). The Pt–DNA levels were approximately threefold lower in the resistant cells ($P < 0.0005$). Pt–DNA adducts were also measured 6 and 24 h after the end of the drug exposure and were expressed relative to immediately after incubation (Figure 2E). About 30–40% of the adducts were removed during this time period without a significant difference between the two cell lines.

### Heat (43°C) effects on Pt accumulation and Pt–DNA adduct formation in EN and ER cells

Figure 2 shows the effect of heat on cellular Pt accumulation and Pt–DNA adduct formation in EN and ER cells. Heat affected the accumulation of cDDP in EN cells only slightly (Figure 2A). Although performing a sign test on paired samples indicated a significant effect of both heat treatments ($P < 0.005$), it is clear that the extent of the enhancement was rather small (factor of 1.22 and 1.38 for 30 and 60 min at 43°C respectively). In the resistant ER cells, on the other hand, heat did have a more pronounced effect on Pt accumulation (Figure 2B). Statistical analysis revealed thermal enhancement of accumulation by factors of 1.49 and 1.85 respectively (30 and 60 min at 43°C, $P < 0.0005$).

Total Pt–DNA adduct formation was significantly enhanced ($P < 0.0005$) immediately after 30- and 60-min heat treatments in both EN (1.7-fold) and ER cells (2.5-fold) (Figure 2C and D). At the 6-h timepoint after cDDP removal, relative Pt–DNA adduct levels in the 30-min-heated cells did not differ from the unheated situation in both cell lines. However, the fraction of adducts remaining in the 60-min-heated EN cells was significantly elevated compared with unheated cells (Figure 2E, $P < 0.005$). At

© Cancer Research Campaign 1997

**Figure 4** Drug accumulation and Pt–DNA adduct formation in $GLC_4$ and $GLC_4$-cDDP cells treated with cDDP alone or combined with 43°C heat. Pt accumulation (**A**; $n$ = 2–7) and Pt–DNA adduct formation (**B**; $n$ = 3–6) in $GLC_4$ and $GLC_4$-cDDP cells after treatment with a cDDP concentration of 25 or 50 μg $ml^{-1}$, and Pt–DNA adducts 6 and 24 h after the end of a 25 μg $ml^{-1}$ drug treatment (**C**, $n$ = 3). Cells were treated at 37°C or combined with 43°C heating. Data shown are the mean ± s.e.m. Statistics (Student's $t$-test on relative effects per experiment): $^*P < 0.05$;$^{**}P < 0.025$

24 h after removal of cDDP, a higher fraction of Pt–DNA adducts remained in the 60-min-heated EN cells than in the unheated cells ($P < 0.05$). For the 60-min-heated ER cells as well as the 30-min-heated EN and ER cells, a trend was observed showing that relatively more damage was remaining than in the unheated cells (Figure 2E), indicating a (small) effect of heat on adduct removal.

### Thermochemosensitization of $GLC_4$ and $GLC_4$-cDDP cells

Figure 3 shows the thermal enhancement of cDDP sensitivity in the human cell lines. $GLC_4$ and $GLC_4$-cDDP cells were equally heat sensitive [30 min at 43°C, survival: $GLC_4$ 38.4% and $GLC_4$-cDDP 30.0% (NS); 60 min at 43°C, survival: $GLC_4$ 5.04% and $GLC_4$-cDDP 7.40% (NS)]. It is clear that extensive cDDP sensitization is caused by 43°C hyperthermia. TERs were 2.48 and 2.95 for 30- and 60-min heating, respectively, of the $GLC_4$ cells. For the resistant $GLC_4$-cDDP cells, TERs of 2.10 and 5.85 were found, resulting in a decrease of the resistance factor from 3.3 at 37°C to 1.5 in the 60-min-heated cells.

### Heat effects (43°C) on Pt accumulation and Pt–DNA adduct formation in $GLC_4$ and $GLC_4$-cDDP cells

Figure 4 shows cellular Pt accumulation, total Pt–DNA adduct formation and Pt–DNA adduct levels 6 and 24 h after cDDP incubation in $GLC_4$ and $GLC_4$-cDDP cells when treated with cDDP at 37°C or combined with hyperthermia. When the data for $GLC_4$ and $GLC_4$-cDDP treated at 37°C were compared, no significant differences between the two cell lines could be detected in the three parameters.

Figure 4A shows that heating cells for 30 or 60 min at 43°C did not significantly alter the cellular Pt accumulation in the $GLC_4$ cells. The accumulation in the resistant cells, however, was increased 1.44- and 2.09-fold ($P < 0.05$) for 30 and 60 min heating, respectively, in cells treated with 50 μg $ml^{-1}$ (Figure 4A). Performing a sign test on all individual accumulation data irrespective of the concentration of cDDP used [i.e. also 5 and 10 μg $ml^{-1}$ (data not shown), 25 and 50 μg $ml^{-1}$ cDDP] showed no significant effect of heat on cDDP accumulation in the $GLC_4$ cells but did reveal significant increases in Pt accumulation in the heated $GLC_4$-cDDP cells ($P < 0.05$). Hence, as observed for the EN/ER cells, hyperthermia has a more pronounced effect on cellular Pt accumulation in the resistant cells than in the sensitive cells.

Pt-DNA adduct formation immediately after cDDP treatment was elevated in both heated $GLC_4$ and $GLC_4$-cDDP cells compared with unheated cells (Figure 4B). In the cDDP-sensitive $GLC_4$ cells adduct formation was increased 1.7- to 2.4-fold when combined with hyperthermia. For the cDDP-resistant subline, significant increases in Pt–DNA adduct formation were found in heated cells when the cells were treated with 25 μg $ml^{-1}$ cDDP (twofold increase, $P < 0.05$). The differences in cells treated with 50 μg $ml^{-1}$ cDDP were not significant. However, performing a sign test on all individual Pt–DNA adduct data, irrespective of the concentration of cDDP used (i.e. also 5 and 10 μg $ml^{-1}$ (data not shown), 25 and 50 μg $ml^{-1}$ cDDP), did reveal an effect of both heat treatments on initial Pt–DNA adduct formation in the cDDP-resistant cells ($P < 0.05$). In addition, Pt–DNA adduct formation in $GLC_4$ and $GLC_4$-cDDP cells was measured using quantitative immunocytochemistry (Meijer et al, 1995). Figure 5 shows typical staining of $GLC_4$ and $GLC_4$-cDDP cells treated with 5 μg $ml^{-1}$ cDDP for 4 h with or without a 60-min 43°C heat treatment. The results using this method again clearly indicate a heat-induced increase in DNA platination. When all concentrations used (5, 10 and 25 μg $ml^{-1}$) were taken together, heat increased Pt–DNA adduct formation by a factor of 2.8 in $GLC_4$ cells and by a factor of 1.72 in $GLC_4$-cDDP cells. Hence, these results are similar to those found in the EN/ER cells: heat increases initial Pt–DNA adduct formation in both cDDP-sensitive and -resistant cells.

For Pt–DNA adduct levels remaining at 6 or 24 h after removal of cDDP, no significant differences were observed for $GLC_4$, whereas for $GLC_4$-cDDP a trend was observed showing that 60 min at 43°C increased the fraction of Pt–DNA adducts

© Cancer Research Campaign 1997

**Figure 5** Immunocytochemical detection of Pt-DNA adduct formation in $GLC_4$ and $GLC_4$-cDDP cells treated with cDDP alone or combined with 43°C heat. Typical photographs of $GLC_4$ (**A** and **C**) and $GLC_4$-cDDP (**B** and **D**) cells treated with 5 μg ml$^{-1}$ cDDP for 4 h at 37°C (**A** and **B**) or combined with 60 min 43°C (**C** and **D**). Quantification of immunostaining of this typical experiment: (**A**) $GLC_4$: 5 μg ml$^{-1}$ cDDP, no heat, 4.40; (**B**) $GLC_4$-cDDP: 5 μg ml$^{-1}$, no heat, 3.74; (**C**) $GLC_4$: 5 μg ml$^{-1}$ cDDP plus heat, 13.09; (**D**) $GLC_4$-cDDP: 5 μg ml$^{-1}$ plus heat, 9.15

remaining in the DNA at 24 h ($P = 0.07$) but not at 6 h after the end of the incubation (Figure 4C).

### Correlations between survival and cDDP-induced damage (all cell lines)

Figure 6 shows the correlations between clonogenic survival after treatment with 0.5 μg ml$^{-1}$ cDDP at 37°C or when combined with 43°C hyperthermia and the various parameters studied. Survival after treatment with 0.5 μg ml$^{-1}$ was chosen as this was measurable even in the most sensitive cells (EN cells heated for 60 min). As a measure for cDDP-induced damage, the cellular Pt accumulation and Pt–DNA adduct formation after treatment with 25 μg ml$^{-1}$ cDDP were used, as this concentration was used for all parameters studied. Although we realize that such a correlation between parameters measured at different concentration levels should be taken with care, it is unavoidable because of the relative insensitivity of the biochemical assays that are available. The data provided in Figure 2 suggest that a linear relationship exists between the various parameters and the concentration of cDDP applied, both at 37°C and when combined with hyperthermia. Therefore, extrapolation of biochemical measurements at this cDDP concentration to concentrations relevant for cytotoxicity is assumed to be valid. When all cell lines are taken into consideration, significant correlations were found for all parameters. However, for Pt accumulation (Figure 6A) and Pt–DNA adduct formation immediately after treatment with cDDP (Figure 6B), $r^2$-values were 0.59 and 0.64 respectively, indicating only weak correlations. The best correlation between survival and cellular damage was found at 6 h after cDDP treatment (Figure 6C); more than 95% of the variation in survival could be related to the variation in absolute amount of Pt–DNA adducts at this timepoint. At the 24-h timepoint (Figure 6D), the correlation was slightly weaker ($r^2 = 0.89$).

### DISCUSSION

In this study the mechanisms underlying cDDP sensitization by hyperthermia in cDDP-resistant cells and their parent counterparts were investigated at the level of drug accumulation, DNA adduct formation and post-treatment adduct processing. Our data reveal that the large overall effect of hyperthermia on cDDP sensitivity may be explained by an accumulation of several different (small) heat effects.

The resistance of the murine (ER) cells seems for a large part to be due to decreased drug accumulation and adduct formation. Neither initial total DNA platination nor initial bifunctional adduct

© Cancer Research Campaign 1997

**Figure 6** Correlation plots of survival after cDDP treatment vs cDDP damage Correlation plots of survival of EN, ER, $GLC_4$ and $GLC_4$-cDDP cells after treatment with 0.5 μg $ml^{-1}$ cDDP at 37°C or combined with 43°C hyperthermia vs the various biochemical parameters studied. Plotted are the cellular Pt accumulation after treatment with 25 μg $ml^{-1}$ cDDP (**A**), the Pt–DNA adduct formation after treatment with 25 μg $ml^{-1}$ cDDP (**B**), the Pt–DNA adduct levels at 6 h after the end of a 25 μg $ml^{-1}$ drug treatment (**C**) and the Pt–DNA adduct levels at 24 h after the end of a 25 μg $ml^{-1}$ drug treatment (**D**)

formation (as determined by immunocytochemistry) were significantly lowered in the resistant $GLC_4$-cDDP cells, in contrast to what was found previously in other resistant sublines derived from the $GLC_4$ cells (Hospers et al, 1988, 1990; Meijer et al, 1990). Individual adducts, however, could differ between the cell lines. Cellular GSH may influence adduct formation, e.g. by quenching the conversion of mono- into (specific) bifunctional adducts (Micetich et al, 1983; Eastman, 1987). As we found significantly higher GSH levels in the cDDP-resistant subline (data not shown), this therefore may have altered the kinetics of formation and removal, as well as the spectrum of bifunctional adducts formed, and by this led to resistance.

Several studies using rodent cell lines have indicated that an important effect of heating cells during or before cDDP treatment is increased accumulation of the drug (Wallner et al, 1986; Mansouri et al, 1989; Eichholtz-Wirth and Hietel, 1990; Los et al, 1993; Takahashi et al, 1993; Ohno et al, 1994). This however was not always found to be the case (Herman et al, 1990) or was found to be true for relatively high heat doses only (Takahashi et al, 1993). The present study indicates that in the murine cDDP-sensitive and -resistant EN and ER cells 43°C heat did increase accumulation of cDDP, although the effect was only marginal in the EN cells. Only two studies using human cell lines have been published so far, reporting either increased drug accumulation for treatment at 40°C (Mann et al, 1988) or no effect of increasing treatment temperature up to 45°C on Pt accumulation in either cDDP-sensitive or -resistant cells (Herman et al, 1988). We found that heat causes no increased Pt accumulation in the cDDP-sensitive $GLC_4$ cells, whereas the Pt accumulation was increased in the resistant subline.

Interestingly, in both sets of cell lines studied here, the effect of 43°C heating on cellular Pt accumulation was higher in the resistant cell lines. In all cases published in which an effect of heat on cDDP accumulation was noticed, the same trend of a (slightly) higher effect of heat on drug accumulation in the cDDP-resistant cells can be observed (Wallner et al, 1986; Mann et al, 1988; Mansouri et al, 1989). One of the major mechanisms of resistance in all these cell lines, as well as in the ER cells (this study), was decreased drug accumulation, thereby indicating that the factor causing the accumulation defect in the resistant cells is sensitive to modulation by hyperthermia. The mechanism by which cDDP enters cells and the alteration that causes decreased accumulation in resistant cells is still unknown. The model proposed by Gately and Howell (1993) that accumulation may occur for approximately 50% by passive diffusion and for 50% by facilitated diffusion through a gated channel can account for most experimental data.

*© Cancer Research Campaign 1997* 

The latter part seems more likely to be altered in resistant cells and so it can be speculated that differences in membrane proteins causing either decreased drug uptake or increased drug efflux could also cause differences in heat sensitivity of cDDP accumulation. Whether this is the case remains to be elucidated.

So, a hyperthermic effect on accumulation is often found. This is however not always the case and its mechanism is unclear. Moreover, enhancement of cDDP toxicity by heating after drug exposure has been shown to be possible in some cell lines and tumours (Fisher and Hahn, 1982; Baba et al, 1989; Urano et al, 1990), indicating that hyperthermia must have other effects that can contribute to cDDP sensitization (see below).

Under all conditions studied, it was found that heat increased the levels of Pt-DNA adducts formed immediately after the drug treatment. The few studies that have measured total DNA platination in heated cells also reported increased overall Pt–DNA adduct levels (Los et al, 1993; Ohno et al, 1994). In our study, this was true also for the cDDP-sensitive cells that showed only minor heat effects on drug accumulation. Theoretically, the latter could be due to a hyperthermia-induced decrease in GSH levels reducing the ability of the cell to detoxify cDDP before reacting with the DNA. However, heating at 43°C decreased GSH levels in the murine cells by only 15% (ER) and 25% (EN) and did not affect GSH levels in the human cells at all (data not shown). Also, heat may have increased the reactivity of cDDP with DNA. In vitro incubation of plasmid DNA with cDDP revealed increased reaction rates at 42°C compared with 37°C (Herman et al, 1989), whereas the reaction of cDDP with salmon sperm DNA in solution showed no temperature dependency (Los et al, 1993). Both experiments, however, do not take into account the complex structure of DNA in the eukaryotic nucleus. This is, of course, most relevant when treating intact cells, and is especially important as it has been shown that hyperthermia causes dramatic changes in chromatin structure (Laszlo, 1992; Kampinga, 1993) that may influence Pt binding to the DNA.

Heat effects on Pt–DNA damage induction in cDDP-resistant cells have not been studied before. In the present study, however, no difference was found between sensitive and resistant cells, indicating that the mechanisms of cDDP-resistance do not interfere with heat effects on adduct formation. In any case, it is clear that hyperthermic potentiation of cDDP toxicity is also, in part, due to elevated levels of initial DNA damage.

As hyperthermia is known to be able to inhibit repair of DNA damage induced by other means [e.g. ionizing radiation (Konings 1987) and UV irradiation (Sakkers et al, 1993)], we also studied the removal of Pt–DNA adducts. The percentage adducts removed was not always found to be significantly decreased in the heated cells. Yet, in all cell lines studied here, a trend was observed that more damage was remaining in the DNA of the heated cells.

Studies on repair of Pt–DNA damage are always complicated by the fact that, after the end of a drug exposure, still additional adducts are formed during the removal of others. Moreover, heat-induced inhibition of repair of DNA damage is to be expected to last for only a few hours. Cells recover within a few hours from the heat damage causing repair inhibition (most likely heat-induced protein aggregation; Konings 1992; Sakkers et al, 1993; Stege et al, 1995). After this time period, repair resumes at normal rates (Sakkers et al, 1993, 1995*a* and *b*). Meyn et al (1980) studied kinetics of interstrand cross-link (ISCL) formation and removal in unheated and 43°C-heated Chinese hamster ovary cells. Their data show that the post-incubation increase in ISCL seems to be elevated and prolonged in heated cells, suggesting that the balance between formation and removal of cross-links is altered as a result of (transient) inhibition of DNA repair. Our data for total DNA platination are suggestive of a similar effect; in the EN and $GLC_4$ cells, in particular, high total DNA platination levels were found at 6 h after incubation in the 60-min-heated cells. In a recent publication by Ohno et al (1994), no effect of heating on the kinetics of ISCL formation in murine L1210 cells was seen; however the temperature used in this study (41.5°C) may have been too low, as such temperature treatments generally do not cause significant nuclear protein aggregation (unpublished data) and repair inhibition of, e.g. radiation-induced strand breaks (Dikomey, 1982). Yet, in another study (Los et al, 1993) no effect of heat (43°C) on the kinetics of total Pt-adduct formation and removal was seen. They, however, used a different immunocytochemical method to assay for DNA platination and two different concentrations of cDDP for heated and unheated cells, impairing a direct comparison between their data and ours. Yet again, besides altered drug accumulation and adduct formation, altered adduct processing (repair) may also contribute to enhanced cDDP lethality under hyperthermic conditions.

In conclusion, like the mechanisms of resistance to cDDP, the effects of hyperthermia on cDDP action seem to be multifactorial. Heat was shown to have some effect on all the parameters studied. A strong argument in favour of the multifactorial effects of hyperthermia are the results shown in Figure 6; the best correlations are found with the Pt-DNA adduct levels at 6 and 24 h after exposure to the drug. These parameters reflect not only heat effects on Pt accumulation and initial Pt-DNA adduct formation but also on the kinetics of Pt-DNA adduct formation and removal. So, although the effect of hyperthermia on some of these individual parameters may not be very large, the combined effect may account for the large potentiation of cDDP action that heat causes. This also explains why single-parameter analyses, as performed in the past, have been inconclusive so far. We have shown that these pleiotropic effects of heat are also effective in enhancing cDDP accumulation, adduct formation and/or adduct removal in cDDP-resistant cells. This indicates why the multifactorial aspects of hyperthermia have been found to be so suitable for the (partial) reversal of cDDP resistance in a variety of cell lines with different mechanisms of resistance (Wallner et al, 1986; Herman et al, 1988; Mansouri et al, 1989; Konings et al, 1993; Hettinga et al, 1994; this study). Therefore, our data provide a scientific basis for supporting the use of combined heat and cDDP chemotherapy in cDDP-resistant tumours, and therefore this combined modality deserves further attention in the clinic.

### ABBREVIATIONS

cDDP, *cis*-diamminedichloroplatinum(II), cisplatin; Pt, platinum; FITC, fluorescein isothiocyanate; RPMI-1640, Roswell Park Memorial Institute medium 1640; PBS, phosphate-buffered saline (1.5 mM potassium dihydrogen phosphate, 6.5 mM disodium hydrogen phosphate, 137 mM sodium chloride, 2.7 mM potassium chloride, pH 7.4); FAAS, flameless atomic absorption spectrometry; ISCL, interstrand cross-links; TER, thermal enhancement ratio – ratio between the cisplatin concentration needed to kill 90% of the cells at 37°C and the concentration of cisplatin needed to kill 90% of the cells when combined with heat treatment; GSH, glutathione.

### ACKNOWLEDGEMENTS

The authors wish to thank Edwin Modderman, Geurt Wolsink and Francien Lode for technical assistance in isolating DNA from

© Cancer Research Campaign 1997

cDDP-treated cells. Jan Ymker and Dries Groefsema (Hospital Pharmacy of the University Hospital Groningen) are gratefully acknowledged for skilful Pt measurements. The authors thank WJ Sluiter PhD for expert help with statistical analysis of the data. This study was financially supported by grant GUKC 91-09 of the Dutch Cancer Society.

### REFERENCES

Andrews PA and Howell SB (1990) Cellular pharmacology of cisplatin: perspectives on mechanisms of acquired resistance. *Cancer Cells* **2**: 35–43

Baba H, Siddik ZH, Strebel FR, Jenkins GN and Bull JMC (1989) Increased therapeutic gain of combined cis-diamminedichloroplatinum(II) and whole body hyperthermia therapy by optimal heat/drug scheduling. *Cancer Res* **49**: 7041–7044

Dikomey E (1982) Effect of hyperthermia at 42°C and 45°C on repair of radiation-induced DNA strand breaks in CHO cells. *Int J Rad Biol* **41**: 603–614

Eastman A (1987) Cross-linking of glutathione to DNA by cancer chemotherapeutic platinum coordination complexes. *Chem Biol Interactions* **61**: 241–248

Eichholtz-Wirth H and Hietel B (1990) Heat sensitization to cisplatin in two cell lines with different drug sensitivities. *Int J Hyperthermia* **6**: 47–55

Engelhardt R (1987) Hyperthermia and drugs. *Recent Results in Cancer Res* **104**: 136–203

Fichtinger-Schepman AMJ, Van Oosterom AT, Lohman PHM and Berends F (1987) Cis-diamminedichloroplatinum(II)-induced DNA adducts in peripheral leukocytes from seven cancer patients: quantitative immunochemical detection of the adduct induction and removal after a single dose of cis-diamminedichloroplatinum(II). *Cancer Res* **47**: 3000–3004

Fisher GH and Hahn GM (1982) Enhancement of cis-platinum(II) diamminedichloride cytotoxicity by hyperthermia. *Natl Cancer Inst Monographs* **61**: 255–257

Gately D and Howell SB (1993) Cellular accumulation of the anticancer agent cisplatin: a review. *Br J Cancer* **67**: 1171–1176

Herman TS, Teicher BA, Cathcart KNS, Kaufmann ME, Lee JB and Lee M-H (1988) Effect of hyperthermia on cis-diamminedichloroplatinum(II) and (Rhodamine 123)$_2$ [tetrachloro-platinum(II)] in a human squamous cell carcinoma line and a cis-diamminedichloroplatinum(II)-resistant subline. *Cancer Res* **48**: 5101–5105

Herman TS, Teicher BA, Chan V, Collins LS and Abrams MJ (1989) Effect of heat on the cytotoxicity and interaction with DNA of a series of platinum complexes. *Int J Rad Oncol Biol Phys* **16**: 443–449

Herman TS, Teicher BA, Pfeffer MR, Khandekar VS and Korbut TT (1990) Interaction with hyperthermia of tetrachloroplatinum(II)(Nile Blue)$_2$ and tetrachloroplatinum(II)(Neutral Red)$_2$ in EMT6 murine cells and the murine FSaIIC fibrosarcoma. *Cancer Res* **50**: 3826–3831

Hettinga JVE, Lemstra W, Meijer C, Mulder NH, Konings AWT, De Vries EGE and Kampinga HH (1994) Hyperthermic potentiation of cisplatin toxicity in a human small cell lung carcinoma cell line and a cisplatin resistant subline. *Int J Hyperthermia* **10**: 795–805

Hospers GAP, Mulder NH, De Jong B, De Ley L, Uges DRA, Fichtinger-Schepman AMJ, Scheper RJ and De Vries EGE (1988) Characterization of a human small cell lung carcinoma cell line with acquired cisplatin resistance *in vitro*. *Cancer Res* **48**: 6803–6807

Hospers GAP, Meijer C, De Leij L, Uges DRA, Mulder NH and De Vries EGE (1990) A study on human small cell lung carcinoma (hSCLC) cell lines with different sensitivities to detect relevant mechanisms of cisplatin (cDDP) resistance. *Int J Cancer* **46**: 138–144

Kampinga HH (1993) Thermotolerance in mammalian cells. Protein denaturation and aggregation, and stress proteins. *J Cell Science* **104**: 11–17

Konings AWT (1987) Effects of heat and radiation on mammalian cells. *Radiat Phys Chem* **30**: 339–349

Konings AWT (1992) Thermal radiosensitization: role of heat shock proteins in heat-induced alterations of protein conformation. In *Proceedings of the 6th Int. Congress on Hyperthermic Oncol*., Gerner EW (ed.), pp. 109–113. Arizona Board of Regents: Tucson, AZ, USA

Konings AWT, Hettinga JVE, Lemstra W, Humphrey GB and Kampinga HH (1993) Sensitizing for cis-diamminedichloroplatinum(II) action by hyperthermia in resistant cells. *Int J Hyperthermia* **9**: 553–562

Laszlo A (1992) The thermoresistant state: protection from initial damage or better repair? *Exp Cell Res* **202**: 519–531

Los G, Van Vugt MJH, Den Engelse L and Pinedo HM (1993) Effects of temperature on the interaction of cisplatin and carboplatin with cellular DNA. *Biochem Pharmacol* **46**: 1229–1237

Mann SC, Andrews PA and Howell SB (1988) Comparison of lipid content, surface membrane fluidity, and temperature dependence of cis-diamminedichloroplatinum(II) accumulation in sensitive and resistant human ovarian carcinoma cells. *Anticancer Res* **8**: 1211–1216

Mansouri A, Henle KJ, Benson AM, Moss AJ and Nagle WA (1989) Characterization of a cisplatin-resistant subline of murine Rif-1 cells and reversal of drug resistance by hyperthermia. *Cancer Res* **49**: 2674–2678

Meijer C, Mulder NH, Hospers GAP, Uges DRA and De Vries EGE (1990) The role of glutathione in resistance to cisplatin in a human small cell lung cancer cell line. *Br J Cancer* **62**: 72–77

Meijer C, De Vries EGE, Dam WA, Wilkinson MHF and Mulder NH (1995) Immunocytochemical detection of DNA-platination in human tissue using a polyclonal antibody: feasibility of predictive testing. *Proc Am Assoc Cancer Res* **36**: 2377

Meyn RE, Corry PM, Fletcher SE and Demetriades M (1980) Thermal enhancement of DNA damage in mammalian cells treated with cis-diamminedichloroplatinum(II). *Cancer Res* **40**: 1136–1139

Micetich K, Zwelling LA and Kohn KW (1983) Quenching of DNA-platinum(II) monoadducts as a possible mechanism of resistance to cis-diamminedichloroplatinum(II) in L1210 cells. *Cancer Res* **43**: 3609–3613

Ohno S, Siddik ZH, Kido Y, Zwelling LA and Bull JMC (1994) Thermal enhancement of drug uptake and DNA adducts as a possible mechanism for the effect of sequencing hyperthermia on cisplatin-induced cytotoxicity in L1210 cells. *Cancer Chemother Pharmacol* **34**: 302–306

Sakkers RJ, Filon AR, Brunsting JF, Kampinga HH, Mullenders LHF and Konings AWT (1993) Heat-shock treatment selectively affects induction and repair of cyclobutane pyrimidine dimers in transcriptionally active genes in ultraviolet-irradiated human fibroblasts. *Radiat Res*, **135**: 343–350

Sakkers RJ, Filon AR, Kampinga HH, Konings AWT and Mullenders LH (1995*a*) Repair of UV-induced pyrimidine(6-4)pyrimidone photoproducts is selectively inhibited in transcriptionally active genes after heat treatment of human fibroblast. *Int J Radiat Biol* **67**: 495–499

Sakkers RJ, Filon AR, Brunsting JF, Kampinga HH, Konings AWT and Mullenders LH (1995*b*) Selective inhibition of repair of active genes by hyperthermia is due to inhibition of global and transcription coupled repair pathways. *Carcinogenesis* **16**: 743–748

Stege GJJ, Kampinga HH and Konings AWT (1995) Heat-induced intranuclear protein aggregation and thermal radiosensitization. *Int J Radiat Biol* **67**: 203–209

Takahashi I, Maehara Y, Kusumoto H, Kohnoe S and Sugimachi K (1993) Heat enhances the cytotoxicity of cis-diamminedichloroplatinum(II) and its analogues cis-1,1-cyclobutane-dicarboxylato(2R)-2-methyl-1,4-butanediammineplatinum(II) and cis-diammine(glycolato)platinum in vitro. *Cancer Chemother Pharmacol* **33**: 31–35

Urano M, Kahn J and Kenton LA (1990) The effect of cis-diamminedichloroplatinum(II) treatment at elevated temperatures on murine fibrosarcoma, FSa-II. *Int J Hyperthermia* **6**: 563–570

Wallner KE, Degregorio MW and Li GC (1986) Hyperthermic potentiation of cis-diamminedichloroplatinum(II) cytotoxicity in chinese hamster ovary cells resistant to the drug. *Cancer Res* **46**: 6242–6245

© Cancer Research Campaign 1997